# Cushing’s disease and bone

**DOI:** 10.1007/s11102-024-01427-7

**Published:** 2024-07-15

**Authors:** Aleksandra Zdrojowy-Wełna, Barbara Stachowska, Marek Bolanowski

**Affiliations:** Department of Endocrinology, Diabetes and Isotope Therapy, Wroclaw Medical University, Wroclaw, Poland

**Keywords:** Cushing’s disease, Osteoporosis, Bone fractures, Hypercortisolism, Bone complications

## Abstract

Bone impairment associated with Cushing’s disease (CD) is a complex disorder, mainly involving deterioration of bone quality and resulting in an increased fracture rate, often despite normal bone mineral density. Bone complications are common in patients with CD at the time of diagnosis but may persist even after successful treatment. There is currently no agreement on the optimal diagnostic methods, thresholds for anti-osteoporotic therapy and its timing in CD. In this review, we summarize the current data on the pathophysiology, diagnostic approach and management of bone complications in CD.

## Introduction

The average time to diagnosis of Cushing’s disease (CD) is 38 months [1]. As a result, patients often have multiple complications at initial presentation, including fractures [2]. Endogenous Cushing’s syndrome (CS) is a rare condition and there are no recommendations for the management of bone complications in this specific group of patients. Hypercortisolism affects bone both, structurally and functionally, which is strongly related to the severity of cortisol excess [3]. Reversal of hypercortisolism is essential to improve bone status, but recent data suggest that fracture risk remains elevated many years after curative treatment [4].

## Epidemiology

In the analysis of the ERCUSYN cohort of 1045 patients with CD, osteoporosis was detected in 22% of lumbar spine and 12% of hip dual-energy X-ray absorptiometry (DXA) scans. Vertebral fractures (VFs) were documented in 25% of the X-ray examinations [5]. Other studies have reported a prevalence of osteoporotic fractures ranging from 16 to 78%, while osteoporosis was reported in 22 to 61% of patients with CD [6–9]. However, many VFs may go unrecognized because patients with CD do not routinely undergo radiological examinations and symptoms may be mild [3]. In 37 patients with CD, 78% had radiologically proven VFs, but only half of these were associated with symptoms such as pain, functional limitation or shortening of stature, and 86% of the fractures were multiple [8].

## Pathophysiology of bone complications in hypercortisolism

### Direct effects of glucocorticoids on bone

Glucocorticoids (GCs) are one of the factors that contribute to proper bone development and maintenance, but even mild hypercortisolism is detrimental to bone [10]. Most studies on the effects of GC on bone have been performed in vitro or in animals, whereas in humans they have been performed in iatrogenic CS. They indicate that the mainstay of glucocorticoid-induced osteoporosis (GIO) is the severe inhibition of bone formation due to the direct action of GC on osteoblasts [11, 12]. It is associated with a transient increase in bone resorption at the onset of hypercortisolism, leading to a rapid increase in fracture risk [13]. Later, the inhibition of osteoblast function predominates in the clinical picture, resulting in a low-turnover state [12]. Bone quality is more compromised than bone mass in patients with iatrogenic CS, and fractures may occur despite normal DXA results [14].

In patients with endogenous CS, these mechanisms are less well understood but are likely to follow a similar pattern. In bone samples from patients with active CD compared to those with non-functioning pituitary adenoma (NFPA), there was a downregulation of genes responsible for osteoblast maturation and function, particularly those involved in collagen synthesis. Growth factors and receptors associated with osteoblastogenesis (such as bone morphogenetic protein 2—BMP2, insulin-like growth factor 1—IGF1) and osteoblast transcription factors (such as Runt-related transcription factor—RUNX2) were also downregulated. There was an increase in the levels of microRNAs (miRNAs) known to suppress osteoblastogenesis, while the antagonists of the Wnt-signaling pathway (Dickkopf 1—Dkkk1 and sclerostin) were upregulated in patients with CD [15]. However, other studies have shown conflicting results, as sclerostin was decreased in patients with active CS (71% CD) and later increased after treatment. The authors concluded that this may be related to altered osteocyte function [16]. It is likely that Wnt signaling plays an important role in bone complications associated with both CD and GIO, but the exact mechanisms should be investigated.

The direct effect of GC on osteoclasts is less clear. In patients with CD (mean duration 2 years), factors stimulating osteoclast maturation and activity, such as Receptor Activator for Nuclear Factor κ B (RANK) and its ligand (RANKL), were downregulated in bone samples, probably due to prolonged inhibition of osteoblast function [15]. This effect may be time-dependent, as anti-resorptive treatment appears to be beneficial in the early stages of iatrogenic hypercortisolism [17].

Osteocyte impairment in GIO has been shown in mice and in vitro studies to be associated with osteocyte autophagy, enlargement of osteocyte lacunae and decreased bone hydration [12, 18]. However, this has not yet been demonstrated in patients with endogenous CS.

There is also some data on the direct effect of adrenocorticotropic hormone (ACTH) on bone. ACTH upregulates vascular endothelial growth factor (VEGF) in bone and probably promotes osteoblast differentiation [19, 20]. It has been suggested that CD may provide better bone preservation than adrenal CS, but the data are inconsistent [9, 21, 22].

### Indirect effects of Cushing’s disease on fracture risk

Endogenous hypercortisolism causes proximal myopathy. Increased fat infiltration in the thigh muscles and reduced muscle strength were found in 28 patients with CD after a long remission, regardless of age, sex and menopausal status [23]. In a longitudinal study, CD-associated muscle dysfunction was significant at diagnosis and only partially improved during 4 years of follow-up observation [24]. In the ERCUSYN cohort, older patients with CD presented with muscle weakness and fractures more frequently than younger patients [25]. Another factor contributing to muscle dysfunction in CD is low IGF1, which persists despite treatment [26].

Excess GC leads to growth hormone deficiency (GHD) through central inhibition of growth hormone (GH) release and peripheral expression of GH receptors [27]. Both endogenous hypercortisolism and GHD lead to “low turnover osteoporosis”, which increases the risk of fracture [28–30]. Another important aspect is GHD caused by CD treatment. Patients with GHD and CD remission had a more significant increase in lumbar BMD after two years of GH therapy than patients with GHD without prior CD [31]. Also, in children with CD, treatment of GHD is probably helpful to prevent later osteoporosis [32]. However, data from a Dutch registry showed that after 6 years of follow-up in patients with GHD, the risk of fracture was increased in a subgroup with prior acromegaly, but not prior CD, compared with NFPA [33]. There are no recommendations for GH replacement therapy in patients with CD, hence the decision needs to be made carefully in each individual patient [28].

Hypercortisolism affects calcium and phosphate metabolism. Exogenous and endogenous GC reduce calcium absorption and increase renal calcium loss [34]. Phosphorus balance is also altered, as shown in 75 patients with active CD who had hypophosphatemia more frequently than controls. Low phosphate levels correlated with urinary cortisol excretion and were reversed after treatment [35]. The potential mechanism could be fibroblast growth factor 23 (FGF23)-stimulated phosphaturia, which has been reported with exogenous GC use [36]. Another important aspect is a probable redistribution of parathyroid hormone (PTH) secretory dynamics, with an increase in the amount of PTH in each pulse and a decreased tonic PTH secretory rate, which has been described in GIO [37]. Therapy with the PTH analogue teriparatide has been beneficial in patients treated with exogenous GC, but there are no data in CD [38–41].

Patients with CD also had lower 25-hydroxyvitamin D [25(OH)D_3_] concentrations than controls, which correlated inversely with urinary cortisol excretion. Six weeks after treatment with high-dose cholecalciferol (150,000 IU) in CD, there was a significant increase in 25(OH)D_3_ levels and improvement in metabolic parameters [42]. Interestingly, patients with CD also had a higher 25(OH)D_3_/24,25(OH)_2_D_3_ ratio compared to controls, indicating reduced 24-hydroxylase activity. The increase in 25(OH)D_3_ concentration after treatment was not correlated with body mass index (BMI) in patients with CD, in contrast to controls. This suggests the influence of endogenous hypercortisolism on vitamin D metabolism [43].

Hypogonadotropic hypogonadism is frequent in active CD. The negative effect of CD on the gonadal axis is related to the intensity of hypercortisolism and is largely reversible following the normalization of cortisol levels [44, 45]. In endogenous CS, hypercortisolism has a greater effect on bone than hypogonadism [8]. Sex steroid replacement therapy is generally recommended in hypogonadism to restore bone health, but there are no specific data in CD [46]. Therefore, the decision needs to be made on an individual basis.

**Fig. 1:**
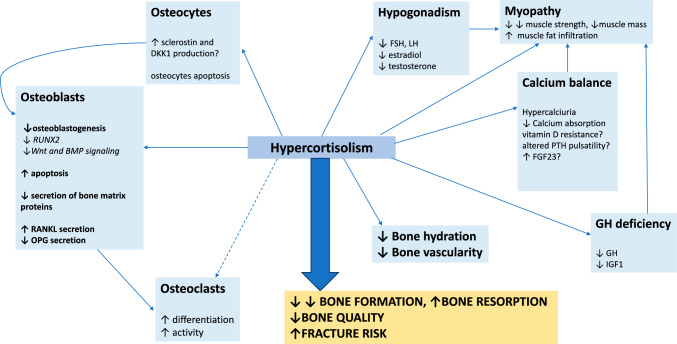
Pathomechanism of bone complications in hypercortisolism. *BMP* bone morphogenetic protein, *DKK*1 dickkopf 1, *FGF*23 fibroblast growth factor 23, *FSH* follicle stimulating hormone, *GH* growth hormone, *IGF*1 insulin-like growth factor 1, *LH* lutropin, *OPG* osteoprotegerin, *PTH* parathormone, *RANKL* Receptor Activator for Nuclear Factor κ B ligand, *RUNX*2 Runt-related transcription factor

The mechanism of GC action on bone is summarized in Fig. 1.

## Methods of bone evaluation in hypercortisolism

It is possible that bone fractures in patients with CD may go unrecognized, as they frequently occur in patients with normal BMD results and without the typical symptoms [8, 47, 48]. The most common locations of fractures are the spine and ribs [3]. The estimated prevalence of these fractures varies considerably, from 16 to 78%. This discrepancy is likely attributable to the severity and duration of hypercortisolism, age, and other risk factors associated with CD (see “Clinical factors influencing bone health in Cushing’s disease” section) [6–9, 19]. Consequently, the screening for fractures in all newly diagnosed patients should be considered, particularly those with abnormal DXA-derived examinations (see below). Vertebral morphometry, including Genant’s semi-quantitative method and algorithm-based qualitative method, as well as plain X-ray, may be employed for the diagnosis of fractures. The low cost and low radiation rate make these methods suitable for use as screening tools. In patients with back pain, magnetic resonance imaging (MRI) is regarded as the gold standard for documenting recent VF, as it may visualize marrow oedema [48].

DXA remains the most common used tool for bone evaluation in both, primary and secondary osteoporosis [3]. In CD, DXA-derived BMD is notably reduced at the lumbar spine, with partial improvement observed after remission [6]. However, bone complications in CD are related particularly to bone quality, which is defined as the properties of bone tissue that contribute to its resistance to fracture [49]. Bone quality may be identified as those intrinsic characteristics of bone tissue that are responsible for its strength and stiffness. The presence of organized, cross-linked collagen fibrils provides a degree of tightness and flexibility, while hydroxyapatite crystals confer stiffness, while remodelling processes in osteons preserve new bone to replace damaged tissue. The balance between these factors is crucial to achieve bone strength [50]. This aspect is not assessed in a DXA examination, and therefore a DXA examination is not sufficient to properly evaluate the risk of fractures in CD [2]. To more accurately assess bone quality, new diagnostic methods have been developed.

One such method is the Trabecular Bone Score (TBS), which is obtained from a computed evaluation of pixel grey-level variations at the lumbar spine DXA. It is highly advantageous in the context of clinical practice, as it necessitates only software modifications to the DXA. TBS provides indirect information on bone microarchitecture [51]. In 19 patients with endogenous CS (74% CD), TBS was observed to be lower than in controls, and a correlation was identified between TBS and disease duration [52]. In contrast to BMD, TBS demonstrated a correlation with the severity of endogenous hypercortisolism, irrespective of its etiology, and exhibited a notable improvement two years following treatment [53]. There are conflicting results regarding the predictive role of DXA-based measurements on fracture risk in CS. In a cross-sectional study comprising 30 patients with active CS (77% with CD), there was a correlation between TBS impairment and VF [54]. In a prospective study comprising 80 middle-aged patients with CS (46% CD), lumbar BMD was identified as the most effective predictor of VF [8]. However, in a younger cohort of 182 patients with CS (83% CD), neither BMD nor TBS remained significant in the final analysis [22]. Therefore, the association between BMD and TBS with the occurrence of fractures in CD is only partly maintained. Low TBS and BMD should provoke an active search for fractures, but VF also occur in patients with normal BMD or TBS values [9, 48, 55].

Another option are CT-based techniques that allow the assessment of trabecular and cortical bone separately. Quantitative CT (QCT) has proven the correlation between hypercortisolism and impairment of vertebral trabecular spine BMD in patients with CS (67% CD) [56]. The more recent method, namely high-resolution peripheral quantitative CT (HR-pQCT) has a spatial resolution that is comparable to that of trabecular bone in vivo, thus enabling the measurement of trabecular thickness [57]. In a group of 30 patients with active CS (73% CD), HR-pQCT detected impaired cortical bone microstructure independently of gonadal status [47]. However, a limitation of HR-pQCT is the restricted field of view, which limits its application to non-central skeletal sites. In contrast, QCT is effectively applied to the proximal femur and lumbar spine [50].

Recently, Giuliodori et al. performed a very interesting analysis of bone mechanical properties in women with long-lasting CS remission (78% of whom had CD). The authors employed a computer-based method, designated as “finite element” (FE) analysis, which utilized QCT scans, but they also performed DXA and TBS examinations. They stimulated a sideway fall, performing FE analysis of mechanical loading, to eventually obtain strain–stress measurements of femoral bone. While all DXA results (including TBS) were similar in patients and controls, QCT-derived results were impaired in patients with CS remission. Moreover, all the strain and stress values indicated higher femoral fracture risk in patients with prior hypercortisolism [4].

Another technique is the measurement of bone marrow fat (BMF) with use of MRI. Hypercortisolism has been demonstrated to increase adiposity of bone marrow, which may result in impairment of osteoblastogenesis [50]. In a group of 20 patients with active CS (80% CD) there was a correlation between BMF and cortisol in blood and urine, as well as an association between BMF and the presence of VF independently of BMD [55].

The role of bone markers assessment in CS is a matter of an ongoing debate. Several studies have demonstrated a negative correlation between osteocalcin and cortisol concentration in individuals with endogenous CS, indicating its potential as a marker of hypercortisolism or even a diagnostic tool [8, 22, 53, 58, 59]. The marker of bone resorption, C-terminal telopeptide of type I collagen (CTX-I), has been observed to exhibit higher levels in patients with endogenous CS in some studies, while in others it has been noted to be lower in comparison to controls [58, 60]. It is likely that this is a time-dependent association, reflecting a rapid increase in bone resorption at the onset of hypercortisolism, followed by a subsequent decline. In the initial cohort of 89 patients with CS (65% CD), the patients exhibited lower bone formation and higher bone resorption markers than the control group. A three- to fourfold increase in bone formation markers was observed during the first year following surgery, with a subsequent moderate decline. The alteration in bone resorption biomarkers was less significant. A T-score improvement was observed in 78% of patients during the two-year follow-up period. These findings demonstrate the importance of considering the timing of bone marker evaluation for accurate interpretation [61]. The data on Wnt-pathway markers are presented in “Direct effects of glucocorticoids on bone” section.

## Clinical factors influencing bone health in Cushing’s disease

The severity of hypercortisolism represents the most significant factor contributing to bone impairment in CD. In a cohort of 182 patients with active CS (83% CD), increased urinary cortisol was the sole predictor of X-ray-confirmed fractures [22]. The duration and severity of endogenous hypercortisolism have been demonstrated to be associated with fractures and BMD in other studies [8, 9].

It is also important to consider the delay to CD diagnosis and the remission time. In patients with endogenous CS (67% CD), the increased fracture risk was observed two years prior to diagnosis [62]. The prospective observation of adolescents with CD demonstrated a significant improvement in BMD results two years after treatment, although the values did not reach those observed in controls [6]. In a further longitudinal prospective study, there was a normalization of lumbar and hip BMD following the curative treatment of endogenous CS (78% CD) after a median follow-up period of almost six years. The improvement in spine BMD occurred earlier than in hip BMD (after 2.5 years), and this was correlated with an increase in osteocalcin levels [63]. In a cross-sectional study comprising 32 females who had undergone CS treatment (78% of whom had CD), impairment of trabecular and cortical bone was observed despite a long remission period (with a mean duration of 10 years). Prior hypercortisolism was found to be independently associated with worse mechanical properties of bone, irrespective of age, physical activity, menopausal status, duration of remission and delay to diagnosis [4]. Furthermore, in a cohort of 419 patients from the Swedish Nationwide Study with long-term CD remission (median 10 years), the standardized incidence ratio of fractures was found to be 1.7-fold increase in comparison to the general population [64]. Conversely, following a six-year period of CD remission, the fracture rate was comparable to that observed in patients undergoing NFPA treatment from the Dutch registry (both groups receiving GHD replacement therapy) [33].

Atraumatic fractures occur despite young age of patients with endogenous hypercortisolemia, but they are more frequent in the elderly [25, 62]. It may be associated with age-related sarcopenia, vitamin D deficiency, a sedentary lifestyle, hypogonadism and other non-communicable diseases that influence bone health. Nevertheless, stronger risk factors (such as the severity of hypercortisolism) have a bigger impact than age itself [8, 22].

In some studies, male sex was associated with an increased risk of fractures, as well as decreased BMD at the lumbar DXA in patients with CS [5, 22, 56], while in others the results were contradictory [62].

It is possible that gonadal status and androgen concentration may play a role in the general risk of fracture, as dehydroepiandrosterone (DHEAS) and testosterone have been found to correlate with bone mineral density (BMD) in patients with endogenous CS [8, 21]. Nevertheless, the influence of hypercortisolism significantly outweighs the protective role of sex steroids [8].

Some authors reported also that the presence of previous fracture was a strong risk factor for the occurrence of a new fracture after the diagnosis of CS [62].

The clinical factors related to bone health in CD are summarized in Table 1.

**Table 1:** Clinical factors influencing bone health in hypercortisolism

Factor	Type of association	References
Severity of hypercortisolism	The severity of hypercortisolism correlated with the fracture risk and BMD	Tauchmanova et al. (2006) [8]
		Trementino et al. (2014) [9]
		Belaya et al. (2015) [22]
Disease duration	The duration of hypercortisolism correlated with fracture risk	Trementino et al. (2014) [9]
Delay to diagnosis	Increased fracture risk 2 years before diagnosis	Vestergaard et al. (2002 [62]
	No influence of delay to diagnosis on bone mechanical properties after remission	Giuliordi et al. (2024) [4]
The resolution of hypercortisolism	Increase of BMD after successful treatment of CS	Di Somma et al. (2003) [6]
		Braun et al. (2020) [61]
		Kristo et al. (2006) [63]
	Impaired bone mechanical properties/fracture risk in CS patients with long-term remission in comparison to healthy population	Giuliordi et al. (2024) [4]
		Papakokkinou E et al. (2020) [64]
	Normalization of fracture risk < 5 years after CS treatment	Vestergaard et al. (2002) [62]
Etiology of CS	Increased risk of osteoporosis and VF in ectopic CS vs pituitary and adrenal CS	Valassi (2022) [5]
		Tauchmanova et al. (2006) [8]
	Higher rate of osteoporosis and fractures in adrenal than pituitary CS	Ohmori et al. (2003) [7]
		Minetto et al. (2004) [21]
	No effect of CS etiology on osteoporosis and fracture rate	Trementino et al. (2014) [9]
		Belaya et al. (2015) [22]
		Vestergaard et al. (2002) [62]
Age	Higher rate of fractures in elderly CS patients	Amodru et al. (2023) [25]
		Vestergaard et al. (2002) [62]
	No effect of age on fracture risk	Tauchmanova et al. (2006) [8]
		Belaya et al. (2015) [22]
		Stachowska et al. (2021) [52]
Sex	Male sex associated with increased fracture rate	Valassi (2022) [5]
		Belaya et al. (2015) [22]
	Male sex associated with lower fracture rate	Vestergaard et al. (2002) [62]
Previous fracture	Previous fracture increased the risk of a new fracture after CS diagnosis	Vestergaard et al. (2002) [62]
Sex steroids	The influence of hypercortisolemia prevails the effect of estrogens on fracture risk	Tauchmanova et al. (2006) [8]
	Androgens have a positive effect on BMD in CS	Ohmori et al. (2003) [7]
		Tauchmanova et al. (2006) [8]
		Minetto et al. (2004) [21]
Growth hormone deficiency	GH replacement therapy in patients after CD treatment improves BMD	Johannson et al. (2004) [31]
		Scommegna et al. (2005) [32]
	No difference in fracture rate between patients with CD remission and GH replacement therapy vs patients after non-functioning pituitary adenoma treatment with GH replacement therapy after 6 years of observation	van Varsseveld et al. (2016) [33]

The numbers in brackets are related to the reference list

*BMD* bone mineral density, *CD* Cushing’s disease, *CS* Cushing’s syndrome, *GH* growth hormone

## Treatment of bone complications in hypercortisolism

There are no clear recommendations for the management of osteoporosis in CD. The treatment of hypercortisolism remains the most important factor in improving bone status. Nevertheless, the guidelines for the treatment of CD suggest that the use of additional anti-osteoporotic medication may be beneficial in accelerating BMD improvement following successful treatment and in preventing further bone loss in patients who have not achieved remission [2].

Some recommendations for fractures prevention in GIO are probably beneficial also in endogenous CS. Given the GC-associated tissue resistance to vitamin D, it is recommended that the 25(OH)D level in the blood be maintained at a value of greater than 32 ng/ml (or 40 ng/ml) [65, 66]. It is recommended that the diet should contain an adequate intake of calcium, at a level of 1,200–2,000 mg per day [67]. It is also recommended that other risk factors for osteoporosis, such as smoking and alcohol consumption exceeding three units per day, should be eliminated [68]. Furthermore, hypercortisolism is associated with an increased risk of falls. Consequently, exercises designed to enhance body balance, strength and resistance training are advised, particularly for individuals over the age of 65 [67].

In the recent guidelines for GIO, oral bisphosphonates (alendronate, risedronate) are recommended in high-risk patients and zoledronate, teriparatide and denosumab in patients with a very high risk of fractures [67]. Two meta-analyses demonstrated that bisphosphonates, teriparatide and denosumab are associated with a decreased odds ratio of VF. Teriparatide and denosumab demonstrated a superior effect over bisphosphonates in this regard [70, 71]. Nevertheless, these recommendations are not directly applicable to endogenous hypercortisolism, as it is difficult to convert GC doses into endogenous CS. Furthermore, GC therapy is recommended for conditions that independently affect bone status.

The intervention studies regarding anti-osteoporotic treatment in endogenous CS are scarce. Bisphosphonates are often regarded as first-line anti-osteoporotic therapy in persistent CS. In a randomized prospective study comprising 39 patients with CD, patients were divided into two groups: one receiving alendronate and one receiving a placebo, while ketoconazole was administered to patients with active disease. In the alendronate-treated group, there was a more pronounced improvement in BMD after one year of treatment compared to the placebo group [72]. Nevertheless, there are some doubts regarding the use of bisphosphonates in CD. Because of the delay in diagnosis, the initial increase in bone resorption is likely to be absent at the time of treatment initiation, which may result in a reduced effectiveness of bisphosphonates [15]. Furthermore, bisphosphonates may potentially influence the anticipated increase in bone turnover following surgery.

Another anti-resorptive drug, denosumab, is characterized by a rapidly reversible action after discontinuation, which may be beneficial in case of CD expecting curative treatment [73]. A substantial body of evidence indicates that denosumab is an efficacious intervention for GIO, demonstrating a greater reduction in VF than oral bisphosphonates [70, 71]. Nevertheless, there is currently no data available regarding its use in CD.

Teriparatide is the first anabolic drug to be approved for the treatment of GIO [74]. Teriparatide activates the Wnt pathway, which makes it a highly beneficial treatment for hypercortisolism [66]. Several prospective studies, with follow-up periods ranging from 18 to 48 months, have demonstrated that teriparatide treatment is more effective than alendronate and denosumab in increasing lumbar and hip BMD and preventing VF in patients with GIO [40, 41, 75, 76]. No studies have been conducted in CD. It is possible that another anabolic drug, romosozumab, may prove beneficial in hypercortisolism, as it targets sclerostin, which is a Wnt-signaling inhibitor. However, the data on sclerostin levels in endogenous CS are contradictory [16, 77]. There is currently no data on the efficacy of romosozumab in CD. Furthermore, the potential cardiovascular risks associated with this drug limit its use in patients with multiple comorbidities.

An additional treatment option is sex hormone replacement therapy and selective estrogen receptor modulator (SERM). The use of these treatments in GIO has been beneficial, although there is currently no data available on their use in CD [78–80]. Furthermore, following the resolution of hypercortisolism, sex steroid levels typically return to normal [45]. There are no recommendations to routinely use sex steroids replacement in GIO, also due to an increased risk of thromboembolic events [46].

## Conclusions

Bone fractures occur in 15–78% of the patients with CD. They are often accompanied by normal BMD results, which may result in them going unrecognised. It is recommended that screening for fractures in CD be considered, particularly in patients with additional risk factors, including severe hypercortisolism, a prolonged delay in diagnosis, low BMD/TBS values, hypogonadism, or advanced age. The diagnostic tools should be focused on bone quality assessment, which is more impaired than bone mass in CD. The prospective studies are needed to evaluate the association between bone examination results, clinical risk factors and fractures in CD. Consequently, a clinical score should be created to stratify fracture risk. The pathogenesis of bone complications in CD is complex, and thus the therapy must be multifactorial. Anabolic medications related to Wnt-signaling appear beneficial from a pathophysiological point of view; however, data concerning their efficacy and safety in CD are lacking. There is an urgent need to conduct prospective studies to establish the therapeutic threshold, optimal bone-active therapy type and its timing in CD.
